# Macrophage Inflammatory Protein-1 Alpha, a Potential Biomarker for Predicting Left Atrial Remodeling in Patients With Atrial Fibrillation

**DOI:** 10.3389/fcvm.2021.784792

**Published:** 2021-12-09

**Authors:** Yung-Lung Chen, Hui-Ting Wang, Pei-Ting Lin, Jiin-Haur Chuang, Ming-Yu Yang

**Affiliations:** ^1^Section of Cardiology, Department of Internal Medicine, Kaohsiung Chang Gung Memorial Hospital, Kaohsiung, Taiwan; ^2^School of Medicine, College of Medicine, Chang Gung University, Taoyuan, Taiwan; ^3^College of Medicine, Graduate Institute of Clinical Medical Sciences, Chang Gung University, Taoyuan, Taiwan; ^4^Emergency Department, Kaohsiung Chang Gung Memorial Hospital, Kaohsiung, Taiwan; ^5^Division of Pediatric Surgery, Department of Surgery, Kaohsiung Chang Gung Memorial Hospital, Kaohsiung, Taiwan; ^6^Department of Otolaryngology, Kaohsiung Chang Gung Memorial Hospital, Kaohsiung, Taiwan

**Keywords:** atrial fibrillation, macrophage inflammatory protein-1 alpha, *RORC*, atrial high-rate episodes, left atrial remodeling

## Abstract

**Objectives:** Left atrial (LA) remodeling itself is an independent risk factor for ischemic stroke and mortality, with or without atrial fibrillation (AF). Macrophage inflammatory protein-1 alpha (MIP-1α) has been reported to be involved in the induction of autoimmune myocarditis and dilated cardiomyopathy. Little is known about whether MIP-1α can be used to predict LA remodeling, especially in patients with AF.

**Methods:** We prospectively enrolled 78 patients who had received a cardiac implantable electronic device due to sick sinus syndrome in order to define AF accurately. AF was diagnosed clinically before enrollment, according to 12-lead electrocardiography (ECG) and 24-h Holter test in 54 (69%) patients. The serum cytokine levels and the mRNA expression levels of peripheral blood leukocytes were checked and echocardiographic study was performed on the same day within 1 week after the patients were enrolled into the study. The 12-lead ECG and 24-h Holter test were performed on the same day of the patients' enrollment, and the device interrogation was performed every 3 months after enrollment. The enrolled patients were clinically followed up for 1 year.

**Results:** There was no difference in baseline characteristics, cytokine levels and mRNA expression between patients with and without AF. Larger LA volume was positively correlated with higher levels of MIP-1α (*r* = 0.461, *p* ≤ 0.001) and the atrial high-rate episodes (AHREs) burden (*r* = 0.593, *p* < 0.001), and negatively correlated with higher levels of transforming growth factor (TGF)-β1 (*r* = −0.271, *p* = 0.047) and TGF-β3 (*r* = −0.279, *p* = 0.041). The higher AHREs burden and MIP-1α level could predict LA volume independently. The mRNA expression of *RORC* was negatively associated with the MIP-1α level.

**Conclusions:** This study showed that higher MIP-1α was significantly associated with LA remodeling and may have the potentials to predict LA remodeling in terms of a larger LA volume, and that circadian gene derangement might affect the expression of MIP-1α.

## Introduction

Atrial fibrillation (AF) is one of the most common cardiac arrhythmias, and it increases the risk of ischemic stroke, systemic embolization, heart failure, and mortality, compared to patients without AF (1). Electrical and structural remodeling play an important role in the pathophysiology of AF development (2). Left atrial (LA) enlargement, an easily-measurable phenomenon, is the default clinical hallmark of structural remodeling that occurs most often in response to LA pressure and volume overload. Changes in LA size, as an index of atrial remodeling, are markers of cardiovascular risk in the general population and also in patients with AF (3–8). There is growing evidence of inflammatory and fibrotic cytokines involvement in the context of both AF occurrence and LA remodeling. Also, it has become clear that inflammation, inflammation-related structural alteration and fibrosis are involved in AF propagation and LA remodeling (9). LA remodeling itself is also an independent risk factor for ischemic stroke and mortality, with or without AF (5). Macrophage-produced cytokines, including interleukin (IL)-l, IL-6, IL-12, and TNF-α, were reported to be associated with atrial fibrosis and AF attacks (10). In addition, several inflammatory and fibrotic cytokines, including IL-1β, IL-6, IL-8, IL-10, tumor necrosis factor (TNF)-α, and transforming growth factor (TGF)-β1, could be used to predict AF occurrence and clinical outcome (10–13). Very few published studies have discussed the predictive biomarkers of LA remodeling in patients with and without AF (9).

Macrophage inflammatory protein-1 alpha (MIP-1α), a kind of chemotactic cytokine secreted by fibroblasts and macrophages, plays a potentially important role in the development of inflammatory responses by recruiting mononuclear cells and modulating cytokine production (14). Increasing evidence suggests that MIP-1α plays a significant role in the etiopathogenesis of cardiovascular disease. A previous study showed that MIP-1α is involved in the induction of autoimmune myocarditis, which is the principal cause of heart failure among young adults and is often a precursor of dilated cardiomyopathy (15). Little is known about whether MIP-1α is associated with LA remodeling and even if it could be used to predict LA remodeling, especially in patients with AF.

*NR1D1* and *ROR* regulates BMAL1 transcription through competing with the ROR response elements at the promoter region of BMAL1, which plays an important role in the maintenance of circadian rhythms in mammals (16). Our previous study showed that *NR1D1* and *RORC* are most significantly and negatively correlated with AF burden and LA remodeling (17). Previous studies also showed those inflammatory and fibrotic cytokines, which were associated with atrial fibrosis and AF episodes, were regulated by *BMAL1* or *NR1D1* (10, 18–22). It is reasonable to speculate that these important circadian clock genes may not only regulate core clock gene expression, but also involved in the expression of inflammatory and fibrotic cytokines that might cause atrial remodeling in patients with AF. Therefore, we hypothesized that *NR1D1* and *RORC* may regulate the expression of macrophage-associated cytokines and fibrotic cytokines, which are associated with a significant change in serum expression of MIP-1α and in LA remodeling in patients with AF.

## Materials and Methods

### Patient Enrollment and Study Protocol

The diagnosis and burden of AF is usually under-estimated in clinical practice. To confirm the diagnosis and burden of AF, in terms of atrial high-rate episodes (AHREs), we prospectively enrolled patients who had received permanent pacemaker implantation due to sick sinus syndrome at our institute from August 2018 through June 2020. The detailed protocol was described in our previous paper (17). In brief, patients with autoimmune disease, malignancy, and acute and chronic inflammation status were excluded from the study. We enrolled patients into this study at least 1 month after the implantation of the pacemaker to prevent the enrollment of those with an inflammatory status post-pacemaker implantation, which may have influenced the expression of inflammatory and fibrotic biomarkers. The 12-lead electrocardiography (ECG) and 24-h Holter test were performed on the same day of the patients' enrollment. The peripheral blood sample and echocardiographic study were performed on the same day within 1 week after the patients were enrolled into the study. The patients' peripheral blood samples were collected between 8:00 and 9:00 a.m., and serum from the peripheral blood of patients were used for analysis of cytokines expression and total leukocytes were used for analysis of *NR1D1, RORC*, and *BMAL1* expression. The enrolled patients were clinically followed up for 1 year. The patient's clinical characteristics, including age, sex, comorbidities such as a history of AF, and echocardiographic findings were analyzed. The study group is defined as patients with AF before the enrollment. The control group is defined as patients who didn't have any AF episode before the enrollment. The definition of heart failure as a baseline characteristic was based on the diagnosis during the previous hospitalization, which included heart failure with a reduced and preserved ejection fraction.

### Definition of AHREs and Measurement of LA Diameter

AF was diagnosed before enrollment by 12-lead ECG or a single-lead ECG recording according to the European Society of Cardiology (ESC) guidelines (1). ECG and 24-h Holter tests of studied patients were reviewed in detail by two electrophysiologists. Cardiac implantable electric device interrogation and reading of intracardiac electrograms stored in the device were also performed by two electrophysiologists every 3 months to confirm the occurrence and duration of AHREs. AHREs were detected by the device automatically and defined as an episode of a fast atrial rate ≥180 beats per minute lasting at least 5 min, according to 2016 ESC guideline (23). The stored intracardiac electrograms were evaluated by the electrophysiologist to exclude artifacts and far-field signals. As described in our previous paper (17), the LA diameter was measured perpendicular to the long axis of the LA posterior wall, inner edge to inner edge, at the level of the aortic sinuses, using a 2-dimensional measure. LA volume was measured using the area-length method using the apical 4- and 2-chamber views at the cardiac cycle of the end-ventricular systolic phase, when the LA is at its maximal size. This study was approved by the Institutional Review Board of Chang Gung Memorial Hospital (IRB number: 201702224B0) and conformed to the guidelines set forth by the Declaration of Helsinki. Written informed consent was obtained from the participants before starting the study.

### Measurement of Inflammatory and Fibrotic Cytokines

Cytokine measurement was performed simultaneously using the Bio-Plex pro human cytokine assay kit and Bio-Rad Bio-Plex 200 multiplex array system (Bio-Rad Laboratories, Hercules, CA, USA). IL-1 receptor antagonist (IL-1Ra), IL-1β, IL-6, IL-8, IL-10, IL-12, IL-18, TNF-α, MIP-1α, MIP-1β, TGF-β1, TGF-β2, and TGF-β3 were measured according to the manufacturer's instructions (24). In brief, 50 μL of beads were added to the well and washed 2 times. Then, 50 μL of plasma sample was added and incubated with antibody-coupled beads for 30 min at room temperature. After washing 3 times to remove unbound materials, the beads were incubated with 25 μL biotinylated detection antibodies for 30 min at room temperature. After washing away the unbound biotinylated antibodies 3 times, the beads were incubated with 50 μL streptavidin-PE for 10 min at room temperature. Following removal of excess streptavidin-PE in three washes, the beads were resuspended in 125 μL of assay buffer. Finally, the beads were read on the Bio-Plex suspension array system, and the data were analyzed using Bio-Plex Manager software (Bio-Rad Laboratories).

### Real-Time Quantitative Reverse Transcriptase-Polymerase Chain Reaction Analysis (qRT-PCR) of *NR1D1, RORC*, and *BMAL1*

Isolation of total peripheral blood leukocytes, RNA extraction, cDNA synthesis, and qRT-PCR were performed as previously described (17). Briefly, the 2 μg RNA input for cDNA synthesis was determined by spectrophotometric OD260 measurement, and cDNA was generated with a High Capacity cDNA Reverse Transcription Kit (Applied Biosystems, Foster City, CA, USA), following the manufacture's protocols. Expression of the *NR1D1* and *RORC* genes was analyzed using the TaqMan system, and all TaqMan Gene Expression Assays were purchased from Applied Biosystems. Expression of the human housekeeping gene, *ACTB (actin beta)*, was used for normalizing *NR1D1* and *RORC* expression in real-time qRT-PCR. Real-time qRT-PCR was performed in an ABI 7500 Fast Real-Time System (Applied Biosystems). The expression levels of the *NR1D1, RORC*, and *BMAL1* were normalized to the internal control *ACTB* to obtain the relative threshold cycle (ΔCt).

### Statistical Analysis

Quantitative data are reported as percentages, median (interquartile range) or mean ± standard deviation as an appropriate approach. The differences in categorical variables between patients with and without AF were analyzed by chi-square or Fisher's exact test, and the differences in continuous data were compared using Student's *t*-test or the Mann–Whitney *U*-test. The AHREs burden was expressed as the ratio of the total AHREs duration to 3 months, as defined by the device automatically. The differences in the expression in the *NR1D1, RORC, and BMAL1* genes between the AF group and the non-AF group using the ΔCt values were analyzed by Student's *t*-test. The folds change in circadian clock genes mRNA expression in patients with and without AF was determined by 2^−ΔΔCt^ calculation. Bivariate correlation analysis was used for variables, including AHREs burden, LA size, and the expression of circadian clock genes (expressed as –ΔCt value) and cytokines. A multiple linear regression model was used to assess the association between dependent variables, LA size, and independent variables, namely age, sex, and cytokines. We used SPSS version 17.0 software (SPSS, Chicago, IL, United States) for all statistical analyses.

## Results

### Baseline Characteristics and Expression of Cytokines, *NR1D1, RORC*, and *BMAL1* Genes of Patients With and Without AF

**Figure 1:**
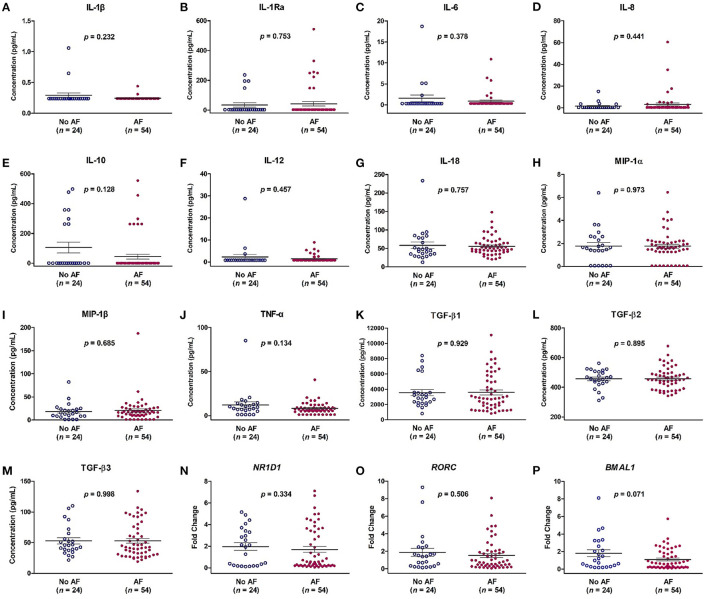
Expression levels of cytokines **(A–M)** and mRNA expression of *NR1D1* **(N)**, *RORC* **(O)**, and *BMAL1* **(P)** in patients with and without atrial fibrillation (AF). The y-axis represents the expression of cytokines or the fold change of the gene expression level in patients with AF compared to patients without AF. The *p*-value analyzed by Student's *t*-test indicates statistical significance as evaluated between patients with (*n* = 54) and without AF (*n* = 24). IL, interleukin; MIP-1α, macrophage inflammatory protein-1 alpha; TGF-β, transforming growth factor beta.

A total of 78 patients were prospectively enrolled. AF was documented in 54 (69%) patients by ECG, 24-h Holter monitoring and pacemaker interrogation data before enrollment. There were 40 (51%) males, and the average age was 70.4 ± 8.2 years. There was no difference in baseline characteristics, including age, sex, hypertension, diabetes mellitus, stroke, heart failure, coronary artery disease, chronic kidney disease, CHA_2_DS_2_-VASc score, and echocardiographic parameters, but only in LA diameter, between patients with and without AF (Table 1). The AHREs burden was higher in the AF group than in the no-AF groups (*p* < 0.001). There was no difference in the percentage of ventricular pacing between patients with and without AF. The LA diameter was larger in the AF group than in the no-AF groups (*p* = 0.011), and the LA volume was also larger in the AF group than in the no-AF groups (*p* = 0.038). There was no difference in the expression level of the 13 cytokines and in mRNA expression of the *NR1D1, RORC*, and *BMAL1* genes between the AF group and the no-AF group (Figure 1).

**Table 1:** Baseline characteristics of the 78-subject study population.

**Variables**	**AF (*n* = 54)**	**No-AF (*n* = 24)**	***p*** **-value**
Age	70.3 ± 8.4	70.7 ± 7.8	0.824
Sex (male/female)	30/24	10/14	0.328
Hypertension	28 (51.9%)	13 (54.2%)	1.000
Diabetes mellitus	11 (20.4%)	7 (29.2%)	0.395
Previous stroke	9 (16.7%)	2 (8.3%)	0.487
Heart failure	6 (11.1%)	2 (8.3%)	1.000
Coronary artery disease	9 (16.7%)	4 (16.7%)	1.000
Chronic kidney disease	4 (7.4%)	4 (16.7%)	0.213
CHA2DS2-VASc score	2.8 ± 1.5	2.8 ± 1.4	1.000
AHREs burden (IQR)	8.1 (1–100)	0 (0–0)	<0.001
Ventricular pacing (%)	4.2 (0.3–37.4)	0.5 (0–8.8)	0.089
**Echocardiographic data**			
LA diameter (mm)	43.7 ± 9.9	39.5 ± 4.6	0.011
LA volume (cm^3^)	76 ± 43	56 ± 23	0.038
Aorta (mm)	32.3 ± 4.6	33.0 ± 4.5	0.519
LVEDD (mm)	46.5 ± 8.8	48.1 ± 8.5	0.456
LVESD (mm)	34.2 ± 8.8	31.2 ± 7.8	0.149
LVEF (%)	61.3 ± 10.3	64.7 ± 8.8	0.162
Septal E/e' ratio	14.3 ± 8.7	14.2 ± 9.9	0.958
DT (ms)	210.0 ± 75.8	193.7 ± 46.3	0.307
PAP (mmHg)	26.9 ± 7.7	25.8 ± 7.4	0.605

*Data are expressed as means ± standard deviation, median (interquartile range) or % (n) as appropriate. AHREs, atrial high-rate episodes; AF, atrial fibrillation; DT, deceleration time; E/e' ratio, the ratio between early mitral inflow velocity and mitral annular early diastolic velocity; IQR, interquartile range; LA, left atrial; LVEDD, left ventricular end-diastolic dimension; LVEF, left ventricular ejection fraction; LVESD, left ventricular end-systolic dimension; PAP, pulmonary artery pressure*.

### Correlation Between LA Size, AHREs Burden, and Cytokines Level in Patients With and Without AF

**Figure 2:**
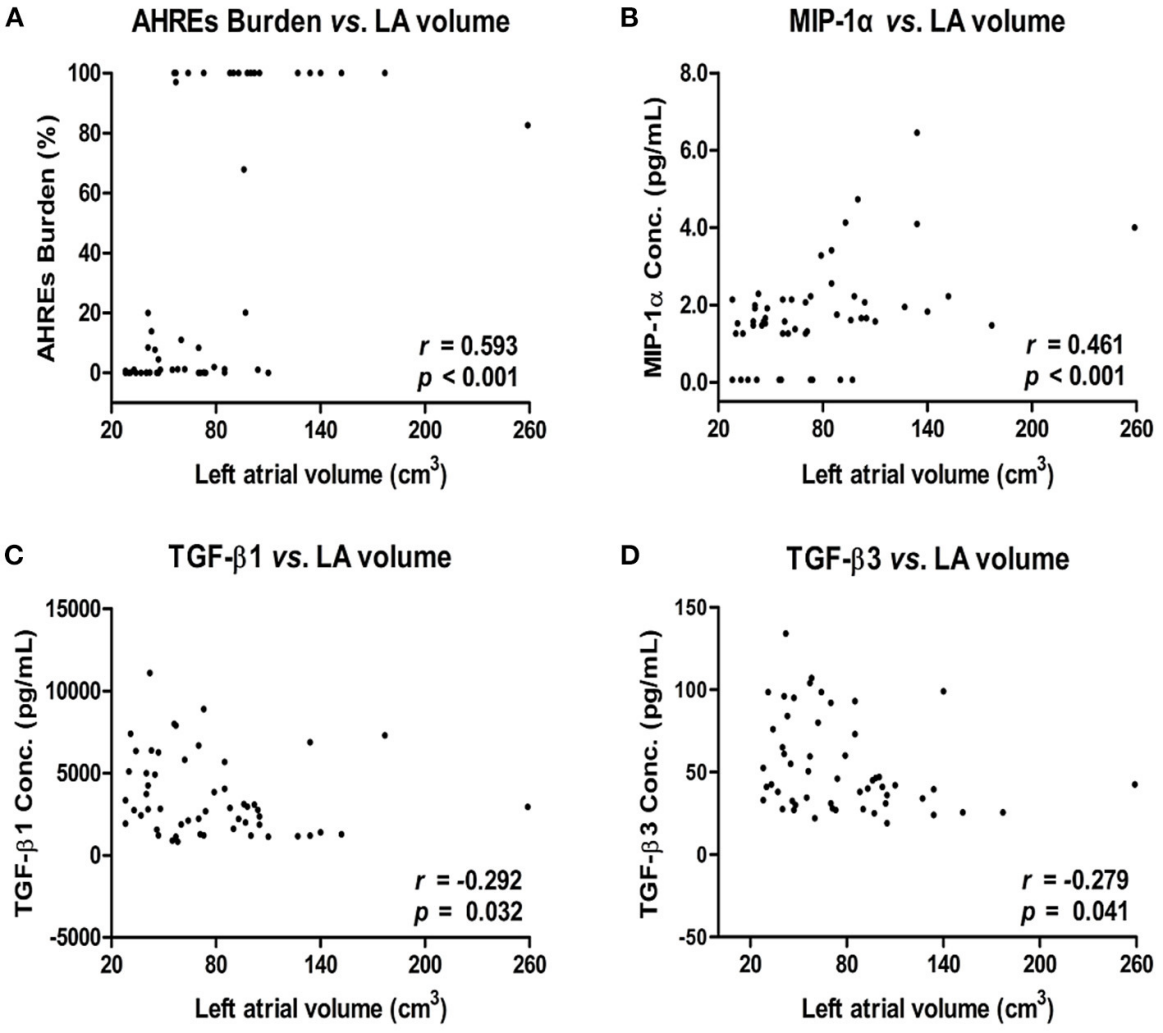
Correlation between left atrial (LA) volume and AHREs burden **(A)** and LA volume and cytokine levels [MIP-1α **(B)**, TGF-β1 **(C)**, and TGF-β3 **(D)**] in patients with AF. The correlation was assessed by bivariate correlation analysis. The *r* and *p*-values indicate the correlation between the expression levels of cytokines and LA volume (expressed as units of cm^3^). AHREs, atrial high-rate episodes; MIP-1α, macrophage inflammatory protein-1 alpha; TGF-β, transforming growth factor beta.

Analysis of the correlation between LA Size, AHREs burden and cytokine levels in patients with AF showed there was a strong and positive correlation between larger LA volume and higher AHREs burden in patients with AF (*r* = 0.593, *p* < 0.001). There was also a strong and positive correlation between larger LA volume and MIP-1α level (*r* = 0.461, *p* < 0.001), and a negative correlation between larger LA volume and TGF-β1 level (*r* = −0.292, *p*= 0.032) and between large LA volume and TGF-β3 level (*r* = −0.279, *p* = 0.041) in patients with AF. There was no significant correlation between LA volume and levels of other cytokines (all *p* > 0.05) (Table 2). The correlation between LA volume and AHREs and between LA volume and cytokines (MIP-1α, TGF-β1, and TGF-β3) is shown in Figure 2. With regard to the association between AHREs and cytokine levels, only MIP-1α level was significantly associated with AHREs burden (*r* = 0.279, *p* = 0.041). No other cytokines were significantly associated with AHREs burden. In addition, although the distribution of age, sex, comorbidities and even CHA_2_DS_2_-VASc scores was the same between patients with and without AF, there was no significant correlation between LA volume and all cytokine levels in those patients without AF (all *p* > 0.05) (Supplementary Table 1).

**Table 2:** Correlation between left atrial (LA) volume and atrial high-rate episodes (AHREs) and between LA volume and cytokines in patients with AF.

**Variables**	***r***	***p*** **-value**
AHREs	0.593	<0.001
MIP-1α	0.461	<0.001
TGF-β1	−0.292	0.032
TGF-β2	−0.263	0.054
TGF-β3	−0.279	0.041
IL-1β	0.228	0.097
IL-1Ra	0.204	0.139
IL-6	−0.076	0.583
IL-8	0.052	0.710
IL-10	0.002	0.986
IL-12	−0.199	0.149
IL-18	0.120	0.388
MIP-1β	0.240	0.081
TNF-α	0.207	0.133

*AHREs, atrial high-rate episodes; IL, interleukin; MIP-1α, macrophage inflammatory protein-1 alpha; TGF-β, transforming growth factor beta*.

### Multiple Linear Regression Model for Assessment of the Association Between LA Size and Other Variables

Variables including age, sex, AHREs, and the 13 cytokines shown in Table 2 were used to assess the association with LA size. The results showed AHREs (Standardized β coefficient = 0.506, *p* ≤ 0.001) and MIP-1α (Standardized β coefficient = 0.556, *p* = 0.014) were significantly associated with LA size (*r*^2^ = 0.582, *p* = 0.002) (Table 3). The study population was then stratified according to quartile distribution on the basis of expression levels of MIP-1α. LA size among these subgroups was analyzed, and showed a significant linear trend of the MIP-1α level (*p* = 0.005) (Table 4). LA size was significantly larger in those patients with MIP-1α > 2.14 pg/ml (quartile 4 group) than in those patients with MIP-1α < 1.27 pg/ml (quartile 1 group) (*p* < 0.01).

**Table 3:** Multiple linear regression model predicting left atrial volume.

**Variables**	**Standardized β coefficient**	***p*** **-value**
Female sex	−0.018	0.901
Age	0.083	0.579
AHREs	0.506	<0.001
MIP-1α	0.556	0.014
TGF-β1	−0.964	0.164
TGF-β2	−0.241	0.350
TGF-β3	1.008	0.197
IL-1β	−0.023	0.856
IL-1Ra	0.035	0.803
IL-6	0.001	0.997
IL-8	−0.061	0.754
IL-10	−0.072	0.705
IL-12	−0.158	0.303
IL-18	−0.183	0.196
MIP-1β	−0.098	0.516
TNF-α	−0.140	0.641

*AHREs, atrial high-rate episodes; IL, interleukin; MIP-1α, macrophage inflammatory protein-1 alpha; TGF-β, transforming growth factor beta*.

**Table 4:** Left atrial volume according to quartile distribution on the basis of MIP-1α level.

**MIP-1α range (ng/mL)**	**Quartile 1 (<1.27)**	**Quartile 2 (1.27–1.64)**	**Quartile 3 (1.64–2.14)**	**Quartile 4 (>2.14)**	***p*** **-value for linear trend**
LA size (cm^3^)	56 ± 22	69 ± 41	78 ± 35	111 ± 55^*^	0.005

*Data are expressed as mean ± standard deviation*.

* *p < 0.01 vs. quartile 1*.

### Correlation of MIP-1α and MRNA Expression of *NR1D1, RORC*, and *BMAL1* Genes in Patients With AF

**Figure 3:**
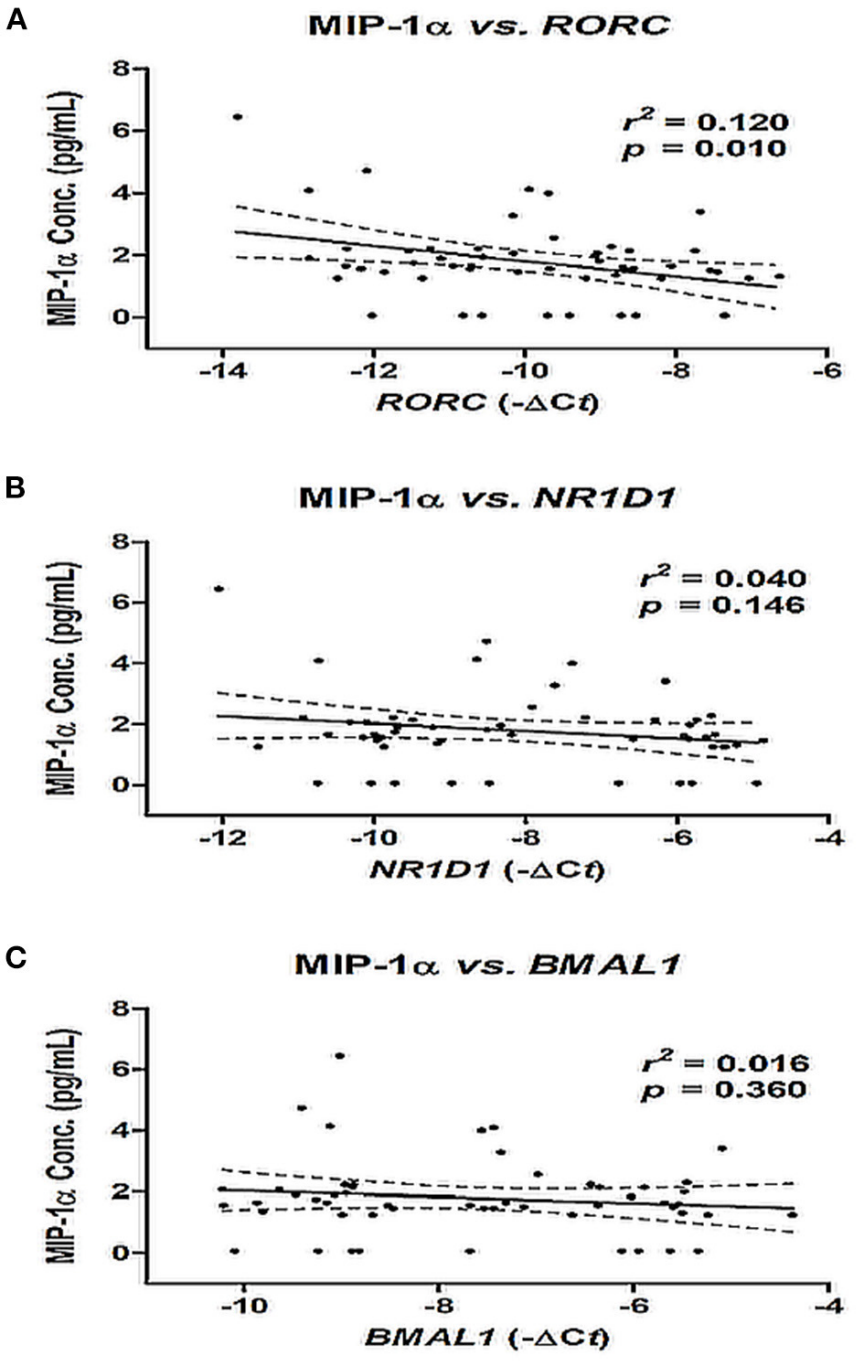
Correlation between MIP-1α level and mRNA expression of *NR1D1* **(A)**, *RORC* **(B)**, and *BMAL1* **(C)** in patients with AF. The correlation was assessed by bivariate correlation analysis. The *r*^2^ and *p*-values indicate the correlation between the MIP-1α level and the mRNA expression of *RORC, NR1D1*, and *BMAL1* genes (expressed as –ΔCt value). MIP-1α, macrophage inflammatory protein-1 alpha.

Bivariate correlation analysis was used to evaluate the relationship between the mRNA expression of *NR1D1, RORC*, and *BMAL1* and the MIP-1α level in patients with AF. There was a negative correlation between *RORC* gene expression and the MIP-1α level (*r*^2^ = 0.120, *p* = 0.010), and there was no significant correlation between the mRNA expression of *NRID*1 and *BMAL1* and the MIP-1α level (both *p* > 0.05) (Figure 3).

## Discussion

There are several important findings in this study. First, LA remodeling in patients with AF was significantly associated with a higher AHREs burden, higher MIP-1α, and lower levels of TGF-β1 and TGF-β3. In contrast, there was no association between LA volume and cytokine levels in patients without AF. Second, among these factors, a higher AHREs burden and higher MIP-1α level could independently predict LA remodeling in patients with AF. Those patients with the highest quartile level of MIP-1α (>2.14 ng/ml) had a larger LA volume than those patients with the lowest quartile level of MIP-1α (<1.27 ng/ml). Third, it is worth noting that mRNA expression of *RORC* only, and not *NR1D1* or *BMAL1*, was significantly and negatively correlated with the MIP-1α level in patients with AF.

MIP-1α is well-known for its role in the activation and migration of leukocytes into areas of inflammation. Previous studies showed MIP-1α was associated with left ventricular remodeling, and even could be used to predict the clinical outcome of patients with atherosclerosis, myocardial ischemia, and heart failure (25–28). The results of our study showed that MIP-1α could significantly predict LA remodeling in patients with AF. During the period of atrial injury due to volume or pressure overload and inflammation, cardiac fibroblasts, or resident cardiac macrophages may secrete MIP-1α, which may cause activation and migration of leukocytes into areas of inflammation and participate in the course of repair (29). The latter suggests that MIP-1α may be involved in atrial fibrosis and remodeling in patients with AF. We also found that the AHREs burden and MIP-1α level could be used to predict larger LA size in patients with AF. In those AF patients with potential LA remodeling, more aggressive echocardiography and even cardiac magnetic resonance imaging follow-up may be indicated to evaluate LA remodeling and fibrosis, and this could be used for risk stratification.

Of interest, the influence of MIP-1α on LA remodeling is significant only in the presence of AF, after adjusting for the CHA_2_DS_2_-VASc score and other demographic data in the present study. Once AF is initiated, the arrhythmia itself causes electrical and structural remodeling, which perpetuates AF and also increases the AF burden (the phenomenon of “AF begets AF”). This may explain both the significant association of higher AHREs burden with LA remodeling and the strong, positive association of MIP-1α level with LA size, and also predict LA remodeling in patients with AF, but not in patients without AF.

TGF-β1 is an important pro-fibrotic biomarker that involves LA fibrosis and remodeling in AF (30). A previous study showed that atrial fibrogenesis is accompanied by a biphasic response of TGF-β1, with an early increase of TGF-β1 in paroxysmal AF patients and a later loss of responsiveness to TGF-β1 in persistent AF (31). The results of our study were in accordance with those of previous studies, in which the TGF-β1 level was negatively correlated with LA size (22, 32). Another study also showed that MIP-1α can induce the expression of TGF-β, and that TGF-β is a potent down-regulator of MIP-1α, which reveals the complex interaction between TGFβ and MIP-1α (33). In our study, we found that MIP-1α rather than TGF-β1 could better predict LA remodeling in AF, after adjusting for age, sex and other cytokines. Future studies are needed to investigate the interaction of MIP-1α and TGF-β1 in the progression of atrial fibrosis and LA remodeling, as well as the role of resident and circulating macrophages and the secreting MIP-1α involved in LA remodeling in patients with AF.

In this study, the expression of *RORC* but not *NR1D1* and *BMAL1* was negatively correlated with the MIP-1α level, and MIP-1α was positively correlated with the AHREs burden and LA remodeling. Taken together, it is possible that circadian clock genes may be helpful in the maintenance of regular cardiac rhythm. The disruption of circadian clock genes expression might affect the expression of MIP-1α, and then lead to the initiation and perpetuation of AF and LA remodeling. Further studies should be conducted to investigate whether disruption of *RORC* would cause an altered expression of MIP-1α and its effect in AF development and LA remodeling.

However, there were two limitations in this study. First, the sample size was too small to evaluate the predictor of LA remodeling and the possible pathophysiologic mechanism. Further larger cohort studies focusing on the causal effects among *RORC*, MIP-1α, and LA remodeling should be performed to confirm the finding. Second, we did not analyze the relationship between medication for AF treatment and its consequent effect on MIP-1α and *RORC* expression, despite the fact that, to date, there are no known drugs used to treat AF that will affect MIP-1α and *RORC*.

## Conclusions

Our study showed that the MIP-1α level was significantly associated with LA remodeling after adjusting for age, sex, and other cytokines and may have the potentials to predict LA remodeling, in terms of increasing LA volume in AF patients. The decreased gene expression of *RORC* was correlated significantly with a higher MIP-1α level and a larger LA size. However, the underlying causal effect between *RORC*, MIP-1α, and LA remodeling should be elucidated in future studies.

## Data Availability Statement

The raw data supporting the conclusions of this article will be made available by the authors, without undue reservation.

## Ethics Statement

The studies involving human participants were reviewed and approved by Institutional Review Board of Chang Gung Memorial Hospital. The patients/participants provided their written informed consent to participate in this study.

## Author Contributions

J-HC and M-YY led in the conception and design of the study, revised the draft of the manuscript, and supervised and validated the clinical work and results. Y-LC collected research data and prepared the draft of the manuscript. H-TW and P-TL performed clinical work and organized the collected data. Y-LC and H-TW performed statistical analysis and drafted the manuscript. All authors have read and agreed to the published version of the manuscript.

## Funding

This study was supported by grants from the Ministry of Science and Technology of Taiwan (MOST 107-2314-B-182A-151 and MOST 108-2314-B-182A-137) and Chang Gung Memorial Hospital (NMRPG8H0211 and NMRPG8J0251).

## Conflict of Interest

The authors declare that the research was conducted in the absence of any commercial or financial relationships that could be construed as a potential conflict of interest.

## Publisher's Note

All claims expressed in this article are solely those of the authors and do not necessarily represent those of their affiliated organizations, or those of the publisher, the editors and the reviewers. Any product that may be evaluated in this article, or claim that may be made by its manufacturer, is not guaranteed or endorsed by the publisher.
