# Supplementary material for: Macrophage Inflammatory Protein-1 Alpha, a Potential Biomarker for Predicting Left Atrial Remodeling in Patients With Atrial Fibrillation
Source: Front Cardiovasc Med. 2021 Dec 9;8:784792. doi: 10.3389/fcvm.2021.784792 (PMC8695724; doi:10.3389/fcvm.2021.784792)
Supplement: Supplementary file 1 [file Table_1.docx]

**Supplemental table 1.** Correlation between left atrial volume and all cytokine levels in patients without AF.

| **Variables** | ***r*** | ***p* Value** |
| --- | --- | --- |
| MIP-1α | -0.44 | 0.837 |
| TGF-β1 | -0.072 | 0.739 |
| TGF-β2 | -0.329 | 0.116 |
| TGF-β3 | -0.077 | 0.721 |
| IL-1β | 0.161 | 0.452 |
| IL-1Ra | 0.140 | 0.515 |
| IL-6 | 0.071 | 0.741 |
| IL-8 | -0.037 | 0.865 |
| IL-10 | 0.343 | 0.100 |
| IL-12 | 0.081 | 0.708 |
| IL-18 | 0.030 | 0.890 |
| MIP-1β | 0.038 | 0.860 |
| TNF-α | -0.179 | 0.402 |

IL= interleukin, MIP-1α= macrophage inflammatory protein-1 alpha, TGF-β= transforming growth factor beta.
